# KATZNCP: a miRNA–disease association prediction model integrating KATZ algorithm and network consistency projection

**DOI:** 10.1186/s12859-023-05365-2

**Published:** 2023-06-02

**Authors:** Min Chen, Yingwei Deng, Zejun Li, Yifan Ye, Ziyi He

**Affiliations:** School of Computer Science and Technology, Hunan Institute of Technology, Hengyang, 421002 China

**Keywords:** miRNA–disease associations, KATZ algorithm, Network consistency projection

## Abstract

**Background:**

Clinical studies have shown that miRNAs are closely related to human health. The study of potential associations between miRNAs and diseases will contribute to a profound understanding of the mechanism of disease development, as well as human disease prevention and treatment. MiRNA–disease associations predicted by computational methods are the best complement to biological experiments.

**Results:**

In this research, a federated computational model KATZNCP was proposed on the basis of the KATZ algorithm and network consistency projection to infer the potential miRNA–disease associations. In KATZNCP, a heterogeneous network was initially constructed by integrating the known miRNA–disease association, integrated miRNA similarities, and integrated disease similarities; then, the KATZ algorithm was implemented in the heterogeneous network to obtain the estimated miRNA–disease prediction scores. Finally, the precise scores were obtained by the network consistency projection method as the final prediction results. KATZNCP achieved the reliable predictive performance in leave-one-out cross-validation (LOOCV) with an AUC value of 0.9325, which was better than the state-of-the-art comparable algorithms. Furthermore, case studies of lung neoplasms and esophageal neoplasms demonstrated the excellent predictive performance of KATZNCP.

**Conclusion:**

A new computational model KATZNCP was proposed for predicting potential miRNA–drug associations based on KATZ and network consistency projections, which can effectively predict the potential miRNA–disease interactions. Therefore, KATZNCP can be used to provide guidance for future experiments.

**Supplementary Information:**

The online version contains supplementary material available at 10.1186/s12859-023-05365-2.

## Background

In recent years, the association of miRNAs with complex human diseases has been a research focus from a wide range of researchers. A large amount of data has been generated in the course of research, and researchers have established a large number of related databases, such as HMDD [1], miR2Disease [2], dbDEMC [3], miRCancer [4], PhenimiR [5], OncomiRDB [6], OncomiRdbB [7], and MiREC [8]. These databases provide a solid data for the study of disease-associated miRNAs, and a large number of computational methods have continuously emerged to predict the association between miRNAs and diseases [9, 10]. The current computable prediction models can be broadly classified into two categories: prediction models driven by network and prediction models based on machine learning. The computational methods for disease-associated miRNA prediction are described from two perspectives.

The prediction model driven by a network is focused on building a network of relationships based on miRNAs, disease, proteins, and environmental factors [11]. From a general biological assumption, “functionally similar miRNAs are likely to be associated with phenotypically similar diseases, and vice versa” [12, 13]. The corresponding algorithm is designed on the basis of the topology of the relational network. Jiang et al. [14] initially proposed a computational model of hypergeometric distribution for predicting the miRNA–disease association methods. The relationship between the regulatory target genes of miRNAs was used to construct miRNA functional similarity networks. In 2010, Jiang et al. [15] proposed an approach based on genomic data integration for predicting miRNA–disease associations. The abovementioned methods performed predictions based on miRNA–target associations. As the false positives of target genes were high, they cannot achieve high predictive performance. Afterward, a series of prediction methods was produced. For example, Xuan et al. [16] proposed a prediction method HDMP based on k most similar neighbors (KNN) based on the hypothesis that miRNAs in the same miRNA family or subcluster may lead to similar diseases [17]. The prediction model was strongly dependent on the miRNA neighbor profile. In addition, Yang et al. [18] and Chen et al. [19] designed new KNN-based disease association ranking algorithms, namely, NBMDA and RKNNMDA. However, the prediction of these models was biased toward miRNAs with multiple known associated diseases.

Considering that global network similarity can improve the prediction accuracy more effectively than local network similarity, many scholars adopted the global similarity approach to make predictions. In 2013, Zhang et al. [20] proposed a method to predict miRNA–disease associations using the network consistency NetCBI. Chen et al. also proposed a series of miRNA–disease association methods by calculating the Tulapras score to obtain consistent network similarity [21–23]. Randomized wandering algorithms with restart were used for miRNA–disease association prediction by many researchers [24]. In 2012, Chen et al. [25] first proposed a random walk association prediction model, RWRMDA, based on global network. This method cannot predict isolated diseases (diseases without any known association) and new miRNAs (miRNAs without any known association). Xuan et al. [26] designed a computational model, namely, MIDP, based on the random walk algorithm. MIDP can travel randomly in the miRNA–disease bidirectional network, thereby allowing for the prediction of isolated diseases. Chen et al. also designed two miRNA–disease prediction models with restart randomized walk algorithms [27, 28]. Luo et al. [29] hypothesized the potential miRNA–disease association by searching for bipartite graph subgraphs and implementing an unbalanced dual random walk algorithm on a heterogeneous network. Most of these methods cannot address the problem of searching for optimal parameters, and their predictions were overly dependent on known miRNA–disease associations.

In recent years, many researchers have attempted to predict miRNA–disease associations from the perspective of graph topology [30]. Chen et al. [31] constructed a heterogeneous map approach to predict the miRNA–disease association in the HGIMDA model. You et al. [32] proposed a pathway-based miRNA–disease association prediction method (PBMDA). Zhao et al. [33] developed a distance-related set-based prediction model (DCSMDA). Zeng et al. [34] proposed a multi-pathway miRNA–disease association prediction method. Chen et al. [35] developed a miRNA–disease association prediction model (BHCN) based on the dichotomous network common neighbors, achieving good prediction results. Zhang et al. [36] and Yu et al. [37] applied the meta-pathway theory to the field of disease-associated miRNA prediction. Many researchers have also achieved good prediction results using the KATZ algorithm [38–40]. The prediction effect of such methods based on the graph theory was also biased for miRNAs with more known associations, and the parameter selection problem of some models remained unsolved.

Recently, the application of the machine learning method in the field of disease-associated miRNA prediction reached highlight [41]. For example, Liu et al. [42] constructed a prediction model (RNSSLFN) based on reliable negative sample selection and improved a single-hidden-layer feedforward neural network. Chen et al. [43] proposed a prediction method (EGBMMDA) using extreme gradient lifters. Zhang et al. [44] designed a deep learning model (VAEMDA) using a variational self-encoder. Li et al. [45] designed a graph autoencoder model (GAEMDA). Liu et al. [46] proposed a deep forest ensemble learning method (DFELMDA) based on self-encoder. Ji et al. [47] designed a self-variational auto-encoder model based on SVAEMDA. Wang et al. [48] and Liu et al. [49] designed the prediction models SAEMDA and SMALF with stacked auto-encoder, respectively. ER et al. [50] improved the miRNA–disease association prediction accuracy by the ensemble similarity information and deep auto-encoders. Peng et al. [51] designed a prediction model EKRRMDA by using ensemble learning and kernel ridge regression. Chen et al. [52] designed a prediction model DBNMDA based on deep-belief network. Xuan et al. [53] constructed a generative adversarial model GMDA using convolutional self-encoders and multilayer convolutional neural networks. Although neural network methods have been applied and have achieved some results in this field, the following problems exist: First, in feature extraction, the rich structural information contained in the heterogeneous biological network is ignored, resulting in low-quality feature representation, thereby leading to overfitting or underfitting; second, as positive and negative samples in training samples are required in most models, selecting negative samples for prediction models constructed on the basis of supervised learning is difficult; third, such models still lack interpretability because of the nonlinear nature of the model architecture.

Semi-supervised learning methods can overcome the limitation of negative samples requirements for training. For example, Chen et al. [54] developed a semi-supervised model RLSMDA based on regularized least squares. Huang et al. [55] constructed a prediction model LRSSLMDA based on Laplace regularized sparse subspace learning. Peng et al. [56] proposed a new information fusion strategy RLSSLP based on the regularization framework. However, these methods cannot be used to set the initial values and select model parameters during optimization iteration.

Matrix factorization was also used to predict disease–miRNA associations [57–60]. For example, Zeng et al. [61] proposed a miRNA–disease association prediction method through a matrix complementation algorithm, which provided a new idea to address problems such as insufficient data on known miRNA–disease associations. Li et al. [62] constructed a prediction model MCMDA by matrix completion algorithm. Based on MCMDA, Chen et al. designed a modified model IMCMDA [63] and NCMCMDA [64]. In addition, a series of improved models have emerged, such as the improved inductive matrix complementary model (IIMCMP) [65], IMDN model with the addition of biased network regularities [66], neural induction matrix complementation method model (NIMGSA) combined with graph auto-encoder and self-attention mechanism [67], matrix complementation algorithm and label passing algorithm model (MCLPMDA) [68], miRTMC model combining the matrix complementation algorithm with kernel parametric regularized linear least squares under non-negative constraints [69], and DLRMC combining matrix complementation algorithm with double Laplace regularization [70]. These improvements enabled the matrix decomposition model to be scalable. The specific implementation and solution were concise. Such improvements can contribute to solving the sparsity of heterogeneous biological data networks. Some limitations can still be found in such methods. First, some of the models proposed initially, such as MCMDA, cannot predict the potential miRNAs associated with the isolated diseases. Second, a local optimal solution was often obtained through the gradient descent method used in the optimization of some algorithms. Thus, further optimization of algorithms must be further explored. Third, the optimal parameter selection problem of many models has not been solved well.

Given the abovementioned ideas from recent literature, a computational model, namely, KATZNCP, was proposed to discover potential miRNA–disease associations in this paper. As for KATZNCP, the known disease–miRNA association information was initially used to calculate the Gaussian kernel spectral similarity between diseases and miRNAs. Then, the semantic interaction network and Gaussian interaction profifile kernel similarity among diseases were integrated to construct an integrated disease similarity network. The functional similarity network and Gaussian kernel spectral similarity among miRNAs were integrated to construct an integrated miRNA similarity network. Afterward, the known disease–miRNA association network, the integrated disease-semantic similarity network, and the integrated miRNA functional similarity network were constructed into a heterogeneous network. The KATZ algorithm was implemented in the heterogeneous network to obtain the initial prediction scores of disease–miRNA associations. Finally, the miRNA–disease associations were refined and predicted by network consistency projection. The high miRNA–disease relationship score obtained by KATZNCP calculations indicated the high likelihood of their association. The KATZNCP model first synthesized disease-miRNA association, disease and miRNA into a heterogeneous network, then implements the KATZ algorithm to collect the best local information in that heterogeneous network. And finally, obtain the global information of these three networks by network space projection. The steps above prevented prediction results biased towards the known miRNAs while keeping the model available to the prediction of isolated diseases and new miRNAs. It grants a notable solution with simple algorithm, single parameter and low time complexity. Solve the problems exist in current state-of-the-art model in a good way.In evaluating the performance of our proposed method, the LOOCV was adopted to verify its pre-performance. The comparison of the four state-of-the-art methods using the same type of data revealed that KATZNCP had an AUC of 0.9325, which was higher than that of the other methods. In addition, the AUCs calculated by the KATZNCP model for the cross-validation of isolated diseases and new miRNAs were 0.8256 and 0.8351, respectively, which further indicated the excellent predictive performance. In validating the actual application of KATZNCP, lung neoplasms and esophageal neoplasms were selected for a case study. The results show that among the top 50 predicted miRNAs, 50 and 47 were confirmed by relevant databases to be associated with lung neoplasms and esophageal neoplasms, respectively. For the case study of isolated diseases, 50 and 49 of the top 50 predicted miRNAs were confirmed by relevant databases to be associated with lung neoplasms and esophageal neoplasms, respectively. The partial miRNAs that were supported by available data for validation were not obtained. Evidence of their association with disease was also found in the latest repertoire of relevant literature, demonstrating the good predictive performance of our model KATZNCP.

## Materials and methods

### Method overview

**Fig. 1:**
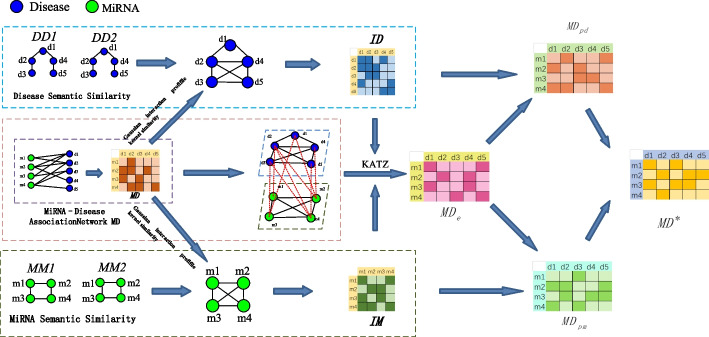
The overall architecture of KATZNCP

In predicting the potential miRNA–disease assocation, a new prediction model KATZNCP was proposed, which consisted of three stages. The detailed inference steps are shown in the flowchart in Fig. 1.

*Step 1* Data preparation. First, the known miRNA–disease association prediction data and the disease semantic similarity data were downloaded from relevant databases. Then, miRNA functional similarity relationships and Gaussian interaction profifile kernel similarity relationships were calculated. Finally, the integrated disease similarity network and integrated miRNA similarity network were constructed.

*Step 2* Association score estimation prediction. Three heterogeneous networks of known miRNA–disease association prediction data, integrated disease similarity network, and integrated miRNA similarity network were constructed as one network. The KATZ algorithm was implemented to obtain the estimated miRNA–disease association prediction scores.

*Step 3* Association score refinement prediction. The integrated disease similarity network was projected into the prediction network. The integrated miRNA similarity network was projected into the prediction network. The two results were weighted to obtain the final miRNA–disease association prediction scores.

### Known miRNA–disease associations

In order to fairly evaluate the performance of the models. Benchmark datasets were employed during the experiments. Specifically, the known miRNA–disease associations dataset was downloaded from HMDD v2.0 (http://www.cuilab.cn/hmdd).As a result, 5430 clinical or experimental verified miRNA–disease associations between 495 miRNAs and 383 diseases were obtained after screening. Detailed associations were represented by a Boolean matrix *MD*, if there is an association between miRNA ${\text{m}}_{{\text{i}}}$ and disease ${\text{d}}_{{\text{j}}}$, corresponding value *MD *(i,j) would be set to 1, otherwise set to 0.

### Semantic similarity calculation of disease

According to the hierarchical information of diseases in MeSH (Medical subject Headings) [1], the relationship between different diseases can be described as a directed acyclic graph (DAG). For any disease *d*, it’s DAG could be represented as *DAG*(*d*) = (*N*(*d*), *E*(*d*)), where *N*(*d*) represents the disease *d*’s ancestor node set (including disease *d* itself), *E*(*d*) represents the related connection. Many scholars use this as a basis to calculate the similarity between diseases. Wang et al. [70] proposed a disease similarity calculation method based on semantic information which accepted an assumption that if two diseases share more disease (common ancestor) entries, the similarity between the two diseases will be greater. At this time, the contribution value of disease $d^{\prime}{\text{s}}$ ancestor node ${\text{d}}_{{\text{a}}}$ to disease *d* was expressed by the following formula:

1 $$D_{d} \left( {d_{a} } \right) = \left\{ {\begin{array}{*{20}l} 1 \hfill & {if\, d_{a} = d} \hfill \\ {\max \left\{ {0.5*D_{d} \left( {d_{a}^{\prime } } \right)|d_{a}^{\prime } \in \,children \,of \,d_{a} } \right\}} \hfill & {if \,d_{a} \ne d} \hfill \\ \end{array} } \right.$$

Based on formula (1), the semantic value *DV*(*d*) of disease *d* was defined as:

2 $$DV\left( d \right) = \mathop \sum \limits_{{d_{a} \in N\left( d \right)}} D_{d} \left( {d_{a} } \right)$$

Finally, the semantic similarity between diseases A and B was constructed as follows:

3 $$DD1\left( {i,j} \right) = \frac{{\mathop \sum \nolimits_{{d_{t} \in N\left( {d_{i} } \right) \cap N\left( {d_{j} } \right)}} D_{{d_{i} }} \left( {d_{t} } \right) + D_{{d_{j} }} \left( {d_{t} } \right)}}{{DV\left( {d_{i} } \right) + DV\left( {d_{j} } \right)}}$$

Named the relationship matrix between diseases calculated by formula 3 as *DD*_*1*._

Xuan et al. [15] proposed another calculation method for calculating the semantic similarity of diseases. This method expresses the contribution value of the disease's ancestor nodes to the disease as follows:

4 $$D_{d} \left( {d_{a} } \right) = - \log \left( {\frac{the\; number\, of\; N\left( d \right)}{{the\; number\; of\; disease}}} \right)$$

Substituting Formula (4) into Formula (2) and Formula (3), named the relationship matrix between diseases calculated as *DD*_*2*_.

### Functional similarity calculation of miRNA

Based on the hypothesis that functionally similar miRNAs were likely to be associated with semantically similar diseases and vice versa, Wang et al. [17] calculated the functional similarity of miRNA through the disease semantic similarity and known miRNA–disease associations. The same method was used to calculate the functional similarity of miRNAs.

For any two miRNAs, the set of diseases associated with them was denoted as two vectors $D^{{\left( {m_{i} } \right)}} = \left\{ {d_{1} ,d_{2} , \ldots ,d_{{m^{\prime } }} } \right\} = \left\{ {d_{{i^{\prime } }} } \right\}_{m} \subset D$ and $D^{{\left( {m_{j} } \right)}} = \left\{ {d_{{1^{\prime \prime } }} ,d_{{2^{\prime \prime } }} , \ldots ,d_{{n^{\prime \prime } }} } \right\} = \left\{ {d_{{j^{\prime \prime } }} } \right\}_{n} \subset D$ The functional similarity of miRNA ${\text{m}}_{{\text{i}}}$ and miRNA ${\text{m}}_{{\text{j}}}$ was calculated as follows:

5 $$mm_{ij} = \frac{{\mathop \sum \nolimits_{{d_{t} \in D^{{\left( {m_{i} } \right)}} }} S\left( {d_{t} ,D^{{\left( {m_{j} } \right)}} } \right) + \mathop \sum \nolimits_{{d_{t} \in D^{{\left( {m_{j} } \right)}} }} S\left( {d_{t} ,D^{{\left( {m_{i} } \right)}} } \right)}}{m + n}$$

where m and n are denoted as the number of diseases associated with miRNA ${\text{m}}_{{\text{i}}}$ and miRNA ${\text{m}}_{{\text{j}}}$, respectively. ${\text{S}}\left( {{\text{d}}_{{{\text{i}}^{\prime } }} ,{\text{D}}^{{\left( {{\text{m}}_{{\text{j}}} } \right)}} } \right)$ represents the degree of association between a given disease ${\text{d}}_{{{\text{i}}^{\prime } }}$ and a given set of diseases $D^{{\left( {m_{j} } \right)}}$. The calculation was as follows:

6 $$S\left( {d_{{i^{\prime } }} ,D^{{\left( {m_{j} } \right)}} } \right) = \mathop {\max }\limits_{{d_{t} \in D^{{\left( {m_{j} } \right)}} }} \left( {dd_{{i^{\prime } t}} } \right)$$

In addition, matrices ${\text{MM}}_{1}$ and ${\text{MM}}_{2}$ were used to denote the miRNA functional similarity matrices obtained by DD_1_ and DD_2_ calculations, respectively.

### Gaussian interaction profifile kernel similarity calculation

Upon measuring the similarity among diseases through the disease semantic similarity, the semantic similarity among various diseases was set as 0 if the data between two diseases were missing. In reducing the impact of this factor on the prediction performance, Gaussian kernel function [71] was applied to the network of association relationships among topologies of bioinformatics nodes. The specific calculation is shown in Eq. (3).

7 $$GD\left( {i,j} \right) = exp\left( { - \gamma_{d} \parallel MD\left( {:,i} \right) - MD\left( {:,j} \right)\parallel^{2} } \right)$$

where $MD\left( {:,i} \right)$ is the *i*-th column of the known miRNA–disease association matrix $MD$. Parameter $\gamma_{d}$ represents the control kernel bandwidth of Gaussian interaction spectrum kernel similarity. It is calculated using the following equation [71]:

8 $$\gamma_{d} = \frac{1}{{\frac{1}{{n_{d} }}\mathop \sum \nolimits_{i = 1}^{{n_{d} }} \left\| {MD\left( {:,i} \right)} \right\|^{2} }}$$

The similarity of the Gaussian interaction spectrum kernel among miRNAs can be calculated using the same method.

9 $$GM\left( {i,j} \right) = exp\left( { - \gamma_{l} \left\| {MD\left( {i,:} \right) - MD\left( {j,:} \right)} \right\| ^{2} } \right)$$

$MD\left( {i,:} \right)$ is the *i-th* row of the matrix $MD^{{n_{m} \times n_{d} }}$. Parameter $\gamma_{1}$ can be obtained by the following equation [71]:

10 $$\gamma_{l} = \frac{1}{{\frac{1}{{n_{m} }}\mathop \sum \nolimits_{i = 1}^{{n_{m} }} \left\| {MD\left( {i,:} \right)} \right\|^{2} }}$$

### Integrated similarity construction

As mentioned previously, the disease semantic similarity, miRNA functional similarity, and miRNA (disease) Gaussian interaction kernel spectral similarity were obtained. By integrating the complementary information from multiple data sources, an integrated similarity approach was used to quantify the similarity of each miRNA (disease) pair, addressing the sparsity of the original similarity matrix. The calculation was as follows:

11 $$ID\left( {i,j} \right) = \left\{ {\begin{array}{*{20}l} {\frac{{DD\left( {i,j} \right) + DD_{2} \left( {i,j} \right)}}{2}} \hfill & {d_{i} and d_{j}\, have \,semantic \,similarity} \hfill \\ {GD\left( {i,j} \right)} \hfill & {otherwise} \hfill \\ \end{array} } \right.$$

12 $$IM\left( {i,j} \right) = \left\{ {\begin{array}{*{20}l} {\frac{{MM_{1} \left( {i,j} \right) + MM_{2} \left( {i,j} \right)}}{2}} \hfill & {m_{i} \,and \,m_{j}\, have \,functional \,similarity} \hfill \\ {GM\left( {i,j} \right)} \hfill & {otherwise} \hfill \\ \end{array} } \right.$$

### Association score estimation prediction

Based on the previously constructed integrated miRNA (disease) similarity, the Katz method was used to obtain the predicted scores estimation of miRNA–disease associations. The Katz method was successfully applied in social network relationship prediction, which calculated the similarity among nodes through the number of walk paths with different step lengths between two nodes. First, a heterogeneous network of miRNA–disease relationships was constructed by using the integrated miRNA–miRNA similarity network, the known miRNA–disease association network, and the integrated disease–disease similarity network. Then, the miRNA–disease associations were predicted on the heterogeneous network using the Katz method. The adjacency matrix of the heterogeneous network was expressed as follows:

13 $${\text{A}} = \left[ {\begin{array}{*{20}c} {IM} & {MD} \\ {MD^{T} } & {ID} \\ \end{array} } \right]$$

Then, the association between miRNAs and diseases was expressed by calculating the number of paths of different lengths among nodes:

14 $${\text{s}}^{{{\text{katz}}}} \left( A \right)_{ij} = \mathop \sum \limits_{l = 1}^{k} \beta^{l} \left( {A^{l} } \right)_{ij}$$

where ${\beta }$ is a non-negative constant used to control the influence of different path lengths, within a range of values $\left( {0,{\text{min}}\left\{ {1,1/{\text{A}}_{2} } \right\}} \right)$. *k* indicates the final maximum path length obtained. When *k* tended to infinity, the above equation can be approximated as follows:

15 $${\text{s}}^{{{\text{katz}}}} = \mathop \sum \limits_{{{\text{l}} > 1}} {\upbeta }^{{\text{l}}} {\text{A}}^{{\text{l}}} = \left( {{\text{I}} - {\beta A}} \right)^{ - 1} - {\text{I}}$$

where *I* is the unit matrix. ${\text{s}}^{katz}$ corresponds to the upper right corner matrix of matrix *A*. $MD_{e}$ is the prediction matrix of miRNA and disease.thus,it have the same structure as *A*(Shown in formula(13)). ${\text{MD}}_{{\text{e}}} { }$ is the prediction matrix of miRNA and disease which is the upper right submatrix of matrix ${\text{s}}^{{{\text{katz}}}}$ that quivalent to the relationship of *MD* with respect to *A*.

### Association score refinement prediction

The accurate prediction scores for miRNA–disease associations calculated by the KATZNCP model consisted of two network-consistent projection scores. One was the spatial projection score of miRNAs and the other was the spatial projection score of diseases. The calculation process was described by calculating the association prediction score between miRNA ${\text{m}}_{{\text{i}}}$ and disease ${\text{d}}_{{\text{j}}}$.

Assuming that the spatial vector formed by the similarity scores of miRNA ${\text{m}}_{{\text{i}}}$ with other miRNAs (including miRNA ${\text{m}}_{{\text{i}}}$ itself) in the integrated miRNA–miRNA similarity network IM was represented as $IM\left( {i,:} \right)$ (the *i*th row of matrix *IM*), the spatial vector formed by miRNAs associated with disease ${\text{d}}_{{\text{j}}}$ in the miRNA–disease predicted score matrix *MD* was represented as $MD_{e} \left( {:,j} \right)$ (the *j*th column of matrix $MD_{e}$). In the miRNA space, the vector ${\text{IM}}\left( {{\text{i}},:} \right)$ represents the relationship between miRNA ${\text{m}}_{{\text{i}}}$ and all miRNAs, the vector ${\text{MD}}_{{\text{e}}} \left( {:,{\text{j}}} \right)$ represents the relationship between diseases ${\text{d}}_{{\text{j}}}$ and all miRNAs. Therefore, the similarity of the variation law could be characterized by the projection of ${\text{IM}}\left( {{\text{i}},:} \right)$ on vector ${\text{MD}}_{{\text{e}}} \left( {:,{\text{j}}} \right)$, which is called as space consistency projection score based on miRNAs. The calculation formula is as shown below:

16 $$MD_{pm} \left( {i,j} \right) = \frac{{IM\left( {i,:} \right) \times MD_{e} \left( {:,j} \right)}}{{MD_{e} \left( {:,j} \right)}}$$

where $MD_{e} \left( {:,j} \right)$ is the two norms of $MD_{e}$.

The consistency projection score based on the disease space can be obtained by using the same method.

17 $$MD_{pd} \left( {i,j} \right) = \frac{{ID\left( {j,:} \right) \times MD_{e}^{T} \left( {:,i} \right)}}{{MD_{e}^{T} \left( {:,i} \right)}}$$

where $MD_{e}^{T} \left( {:,j} \right)$ is the two norms of ${ } MD_{e}^{T}$.

Finally, the miRNA space consistency projection score and disease space consistency projection score were integrated by using Eq. (13) to form the final prediction score.

18 $$MD^{*} = \frac{{MD_{pm} + MD_{pd}^{T} }}{2}.$$

## Results

### Evaluation metrics

In order to systematically evaluate the performance of KATZNCP as well as other comparative methods, A leave-one-out cross-validation (LOOCV) was employed to test the predictive performance of the model. Specifically, one miRNA–disease association was selected as a test sample and all other miRNA–disease associations were regarded as training samples. Repeat these procedure until all miRNA–disease associations were used as a test sample once. The prediction effect was expressed by the receiver operating characteristic (ROC) curve, and the accuracy was quantified by the area under the ROC curve (AUC).ROC curve is a comprehensive indicator reflecting sensitivity (Sensitivity) and specificity (Specificity). The ROC curve reveals the relationship between sensitivity and specificity in a graphical way. By setting different thresholds, a series of corresponding sensitivities and specificities are calculated. Then draw a curve with the true positive rate (True positive rate, TPR, sensitivity or sensitivity) as the vertical axis and the false positive rate (False positive rate, FPR or 1-Specificity) as the horizontal axis. The calculation methods of TPR and FPR are as follows:

19 $${\text{TPR}} = \frac{{{\text{TP}}}}{{{\text{TP}} + {\text{FN}}}}$$

20 $${\text{FPR}} = \frac{{{\text{FP}}}}{{{\text{FP}} + {\text{TN}}}}$$

which TP (True Positive) refer to the number of positive samples that are correctly predicted, that is, the number of positive samples that are predicted as positive samples; FP (False Positive) refer to the number of positive samples that are incorrectly predicted, that is,the number of negative samples predicted as positive samples; TN (True Negative) refer to the number of negative samples correctly predicted, that is, the number of negative samples predicted as negative samples; FN (False Negative) refer to The number of mispredicted negative samples, that is, the number of positive samples that were predicted as negative samples. Considering that we have no confirmed negative samples, we used an alternative.First obtain the upper and lower bounds of the threshold according to the prediction results.Then determine a set of thresholds accordingly. For any certain threshold, if the predicted value is greater than the threshold, the prediction will be considered as positive, otherwise the forecast will be considered as negative.

### Effect of parameter selection

**Fig. 2:**
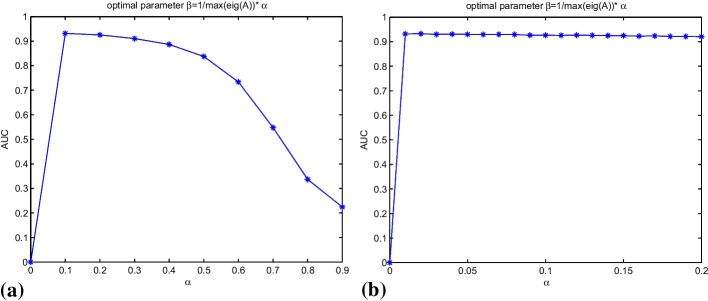
**a** the value of the AUC when ${\upalpha }$ was increased from 0 to 0.9. **b** the value of the AUC when ${\upalpha }$ was increased from 0 to 0.2

In equation ${\text{s}}^{{{\text{katz}}}} = \mathop \sum \limits_{{{\text{l}} > 1}} {\upbeta }^{{\text{l}}} {\text{A}}^{{\text{l}}} = \left( {{\text{I}} - {\beta A}} \right)^{ - 1} - {\text{I}}$, the value of parameter $\beta$ was associated with the prediction effects. In ensuring the convergence of the series, the value of $\beta$ shall be smaller than the inverse of the maximum eigenvalue of the adjacency matrix *A*. In obtaining the optimal parameter $\beta$, $\beta { }$ was set to ${ }\beta = \alpha \times 1/eigA$ (*eigA* was the maximum characteristic root of matrix *A*). Then, with steps of 0.1 and increment of ${\upalpha }$ from 0 to 0.9, 10 LOOCV were to calculate the AUC values. The experimental results obtained by implementing LOOCV are shown in Fig. 2a The results showed that when ${\upalpha }$ = 0, the equation was degenerated to ${\text{s}}^{{{\text{katz}}}} = 0$, indicating that KATZNCP had no prediction capability. When ${\upalpha }$ was increased from 0.1 to 0.9, AUC gradually decreased. AUC reached the maximum at 0.9316 when ${\upalpha }$ was 0.1, followed by 0.9299 when ${\upalpha } = 0.2$. Then, the steps were taken as 0.01 to obtain more accurate weighting parameters. ${\upalpha }$ was gradually increased from 0 to 0.2. Then, LOOCV was performed again. The obtained results are shown in Fig. 2b. The calculated AUC values fluctuated from 0.9299 to 0.9316. When ${\upalpha }$ ranged between 0.01 and 0.05, AUC fluctuated to approximately 0.9320. AUC reached the maximum at 0.9325 when ${\upalpha }$ was 0.02. When ${\upalpha }$ gradually increased from 0.05 to 0.2, the AUC value gradually decreased from 0.9316 to 0.9299. Therefore, 0.02 was finally selected as the value of ${\upalpha }$.

### Comparison with state-of-the-art methods

**Fig. 3:**
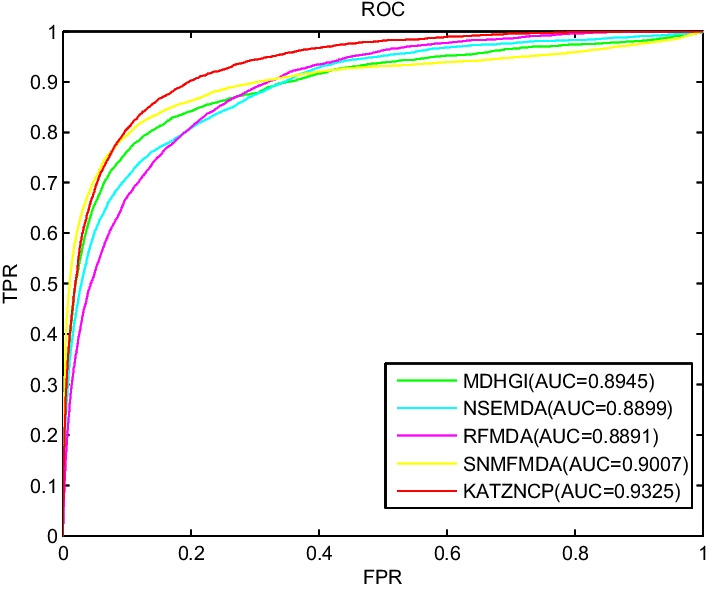
ROC curves of five competitive methods

Similar to the data resources used by KATZNCP, prediction models with excellent prediction results consisted of MDHGI [72], NSEMDA [73], RFMDA [74], and SNMFMDA [75]. These methods were selected for comparison with KATZNCP. Figure 3 shows the LOOCV results of each model, with AUC values of 0.8945, 0.8899, 0.8891, 0.9007, and 0.9325 for MDHGI, NSEMDA, RFMDA, SNMFMDA, and KATZNCP, respectively. KATZNCP showed the best prediction results, which was 4.25%, 4.79%, 4.88%, and 3.53% higher than MDHGI, NSEMDA, RFMDA, and SNMFMDA, respectively. Therefore, the prediction ability of KATZNCP was better than that of MDHGI and other models.

### Validation of new miRNAs and isolated disease prediction capabilities

New miRNAs refer to miRNAs with unknown association information with disease. With the continuous improvement of miRNA recognition techniques, an increasing number of miRNAs were being identified. Inspired by Liang et al. [76], here, another assessment metric was adopted to evaluate the predictive power of the model for new miRNAs, namely, leave one miRNA out cross validations (LOMOCV). In particular, one miRNA was selected as the test sample at one time. All diseases associated with this miRNA were removed before testing. Then, all candidate diseases were prioritized by using the information from other miRNA-associated diseases only, until all miRNAs had been validated as predicted samples.

Isolated diseases refer to diseases with unknown association information with miRNAs. Similar to the simulation of new miRNAs, all its associated miRNAs were removed for each isolated disease to simulate isolated diseases. All candidate miRNAs were prioritized by using the information from other disease-associated miRNAs, which is known as leave one disease out cross validations (LODOCV).

**Fig. 4:**
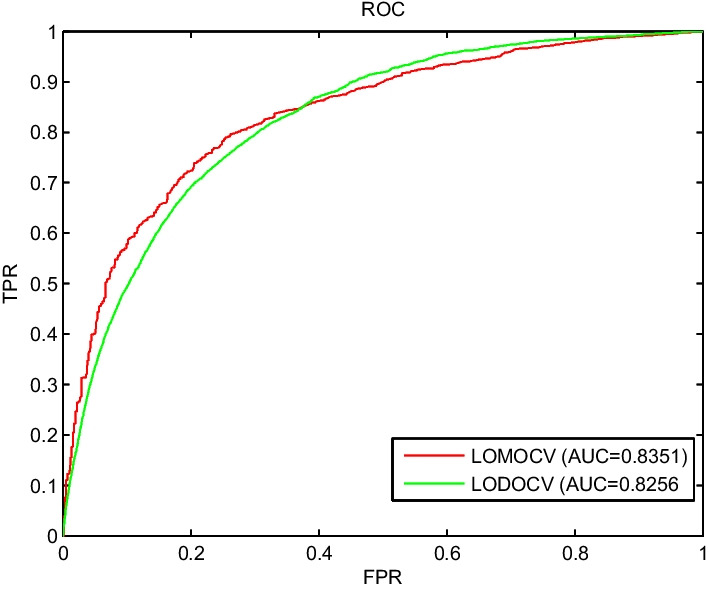
Results of KATZNCP for newmiRNAs and isolated diseases

As shown in Fig. 4, the AUC of KATZNCP was 0.8256 under the LODOCV framework and 0.8351 under the LOMOCV framework.

### Case study

In demonstrating the predictive capability of our proposed model KATZNCP for disease-associated miRNA, two diseases, namely, lung neoplasms and esophageal neoplasms, were selected for case studies. All the prediction results were validated in the two independent databases, namely, HMDD v3.2 [77] and dbDEMC 2.0 [78].

Lung neoplasm is a kind of malignant tumor with rapid progression and poor prognosis. Distant metastasis often occurred, which then led to death. The detection rate of this disease in the early stage was not high, which posed a great threat to people’s health [79]. The prediction of miRNA associated with lung neoplasms was of great practical significance. For lung neoplasms, the top 50 miRNAs related to lung neoplasms predicted by KATZNCP have been supported in two data sets, namely, HMDD v3.2 and dbDEMC (Table 1).

**Table 1:** The top 50 lung neoplasm-related miRNAs

Rank	miRNA name	Evidences	Rank	miRNA name	Evidences
1	hsa-mir-16	HMDD, dbDEMC	26	hsa-mir-152	HMDD, dbDEMC
2	hsa-mir-151a	dbDEMC	27	hsa-mir-194	HMDD,dbDEMC
3	hsa-mir-130a	HMDD, dbDEMC	28	hsa-mir-215	HMDD, dbDEMC
4	hsa-mir-302b	HMDD, dbDEMC	29	hsa-mir-92b	dbDEMC
5	hsa-mir-708	HMDD, dbDEMC	30	hsa-mir-367	dbDEMC
6	hsa-mir-193b	dbDEMC	31	hsa-mir-129	HMDD, dbDEMC
7	hsa-mir-99a	HMDD, dbDEMC	32	hsa-mir-302d	dbDEMC
8	hsa-mir-429	dbDEMC	33	hsa-mir-449a	dbDEMC
9	hsa-mir-149	HMDD, dbDEMC	34	hsa-mir-23b	dbDEMC
10	hsa-mir-302c	dbDEMC	35	hsa-mir-328	HMDD, dbDEMC
11	hsa-mir-106b	HMDD, dbDEMC	36	hsa-mir-320a	dbDEMC
12	hsa-mir-141	HMDD, dbDEMC	37	hsa-mir-345	dbDEMC
13	hsa-mir-451a	HMDD, dbDEMC	38	hsa-mir-153	HMDD, dbDEMC
14	hsa-mir-625	dbDEMC	39	hsa-mir-452	dbDEMC
15	hsa-mir-15b	dbDEMC	40	hsa-mir-130b	HMDD, dbDEMC
16	hsa-mir-195	HMDD, dbDEMC	41	hsa-mir-339	dbDEMC
17	hsa-mir-15a	HMDD, dbDEMC	42	hsa-mir-372	HMDD, dbDEMC
18	hsa-mir-378a	dbDEMC	43	hsa-mir-196b	HMDD, dbDEMC
19	hsa-mir-296	dbDEMC	44	hsa-mir-370	dbDEMC
20	hsa-mir-373	HMDD, dbDEMC	45	hsa-mir-342	HMDD, dbDEMC
21	hsa-mir-20b	dbDEMC	46	hsa-mir-449b	dbDEMC
22	hsa-mir-139	HMDD, dbDEMC	47	hsa-mir-122	HMDD, dbDEMC
23	hsa-mir-204	dbDEMC	48	hsa-mir-99b	dbDEMC
24	hsa-mir-10a	dbDEMC	49	hsa-mir-151b	dbDEMC
25	hsa-mir-302a	dbDEMC	50	hsa-mir-211	dbDEMC

Esophageal neoplasm is the eighth most common cancer worldwide. The effectiveness of treatment for esophageal cancer was largely dependent on its cause [80]. For esophageal neoplasms, among the predicted top 50 miRNAs, 47 miRNAs have been supported in two data sets, namely, HMDD v3.2 and dbDEMC (Table 2). Only the supporting evidence of hsa-mir-200b, hsa-mir-302b, and hsa-mir-302c cannot be found. However, evidence of the association between hsa-mir-200b and esophageal neoplasms was found after searching other literature manually. For example, S. Kirkilevsky [81] found that the expression of miRNA-200b and ERCC1 in EC cells can be used to predict the aggressiveness of esophageal cancer, which was published in 2020. Yang et al. [18] predicted the relationship between hsa-mir-302b and esophageal neoplasms through computational method. The predictive power of KATZNCP was further confirmed by the aforementioned evidence. Although no current medical trials have shown that the two miRNAs, hsa-mir-302b and hsa-mir-302c, were related to esophageal neoplasms, biologists will conduct further experiments to uncover their potential relationship.

**Table 2:** The top 50 Esophageal Neoplasms-related miRNAs

Rank	miRNA name	Evidences	Rank	miRNA name	Evidences
1	hsa-mir-218	dbDEMC	26	hsa-mir-222	dbDEMC
2	hsa-mir-10b	HMDD, dbDEMC	27	hsa-mir-7	HMDD, dbDEMC
3	hsa-mir-200b	Unconfirmed	28	hsa-mir-224	dbDEMC
4	hsa-mir-18b	HMDD, dbDEMC	29	hsa-mir-429	dbDEMC
5	hsa-mir-107	dbDEMC	30	hsa-mir-146b	dbDEMC
6	hsa-mir-127	dbDEMC	31	hsa-mir-497	dbDEMC
7	hsa-let-7f	dbDEMC	32	hsa-mir-221	dbDEMC
8	hsa-let-7d	dbDEMC	33	hsa-mir-17	dbDEMC
9	hsa-mir-125b	HMDD, dbDEMC	34	hsa-mir-30c	dbDEMC
10	hsa-let-7g	dbDEMC	35	hsa-mir-302c	Unconfirmed
11	hsa-mir-135a	dbDEMC	36	hsa-mir-24	dbDEMC
12	hsa-mir-142	dbDEMC	37	hsa-mir-181b	dbDEMC
13	hsa-let-7i	dbDEMC	38	hsa-mir-151a	HMDD, dbDEMC
14	hsa-mir-16	dbDEMC	39	hsa-mir-629	dbDEMC
15	hsa-let-7e	dbDEMC	40	hsa-mir-181a	dbDEMC
16	hsa-mir-18a	dbDEMC	41	hsa-mir-93	HMDD, dbDEMC
17	hsa-mir-124	HMDD, dbDEMC	42	hsa-mir-15b	dbDEMC
18	hsa-mir-133b	dbDEMC	43	hsa-mir-195	dbDEMC
19	hsa-mir-182	HMDD, dbDEMC	44	hsa-mir-1	dbDEMC
20	hsa-mir-302b	Unconfirmed	45	hsa-mir-139	HMDD, dbDEMC
21	hsa-mir-199b	dbDEMC	46	hsa-mir-708	dbDEMC
22	hsa-mir-125a	dbDEMC	47	hsa-mir-338	dbDEMC
23	hsa-mir-9	dbDEMC	48	hsa-mir-138	dbDEMC
24	hsa-mir-106a	dbDEMC	49	hsa-mir-193b	dbDEMC
25	hsa-mir-191	dbDEMC	50	hsa-mir-194	HMDD, dbDEMC

In testing the predictive performance of KATZNCP for isolated diseases, isolated diseases were simulated by the same approach as that of LODCV. Alternatively, all miRNAs associated with the disease to be verified were deleted before KATZNCP was implemented. For lung neoplasm, 132 known associations between lung neoplasm and miRNAs were deleted. KATZNCP was used to predict the potential associations between miRNAs and lung neoplasm. All of the top 50 predicted miRNAs can be supported in HDMM3.2 and dbDEMC databases (Table 3). For esophageal neoplasms, 74 known associations were deleted, and KATZNCP was used for prediction. Of the top 50 predicted associations, 49 were supported in the databases HDMM3.2 and dbDEMC (Table 4). Only hsa-mir-200b was not demonstrated by either database. However, based on previous case analysis of common disease prediction, available studies showed a close relationship between hsa-mir-200b and esophageal neoplasms.

**Table 3:** The top 50 lung neoplasms-related miRNAs candidates predicted by KATZNCP with removed all known lung neoplasms-miRNAs associations and the confirmation of these associations

Rank	miRNA name	Evidences	Rank	miRNA name	Evidences
1	hsa-mir-21	HMDD, dbDEMC	26	hsa-mir-34c	HMDD, dbDEMC
2	hsa-mir-155	HMDD, dbDEMC	27	hsa-mir-182	HMDD, dbDEMC
3	hsa-mir-146a	HMDD, dbDEMC	28	hsa-mir-218	HMDD, dbDEMC
4	hsa-mir-126	HMDD, dbDEMC	29	hsa-mir-210	HMDD, dbDEMC
5	hsa-mir-145	HMDD, dbDEMC	30	hsa-mir-133a	HMDD, dbDEMC
6	hsa-mir-125b	HMDD, dbDEMC	31	hsa-mir-34b	HMDD, dbDEMC
7	hsa-mir-34a	HMDD, dbDEMC	32	hsa-mir-205	HMDD, dbDEMC
8	hsa-mir-221	HMDD, dbDEMC	33	hsa-mir-146b	HMDD, dbDEMC
9	hsa-mir-16	HMDD, dbDEMC	34	hsa-mir-124	HMDD, dbDEMC
10	hsa-mir-200b	HMDD, dbDEMC	35	hsa-mir-200a	HMDD, dbDEMC
11	hsa-mir-200c	HMDD, dbDEMC	36	hsa-mir-148a	HMDD, dbDEMC
12	hsa-mir-20a	HMDD, dbDEMC	37	hsa-mir-183	HMDD, dbDEMC
13	hsa-mir-29a	HMDD, dbDEMC	38	hsa-mir-223	HMDD, dbDEMC
14	hsa-mir-122	HMDD, dbDEMC	39	hsa-let-7b	HMDD, dbDEMC
15	hsa-mir-17	HMDD, dbDEMC	40	hsa-mir-101	HMDD, dbDEMC
16	hsa-mir-199a	HMDD, dbDEMC	41	hsa-mir-18a	HMDD, dbDEMC
17	hsa-mir-196a	HMDD, dbDEMC	42	hsa-mir-181a	HMDD, dbDEMC
18	hsa-let-7a	HMDD, dbDEMC	43	hsa-mir-92a	HMDD, dbDEMC
19	hsa-mir-222	HMDD, dbDEMC	44	hsa-mir-214	HMDD, dbDEMC
20	hsa-mir-1	HMDD, dbDEMC	45	hsa-mir-9	HMDD, dbDEMC
21	hsa-mir-29b	HMDD, dbDEMC	46	hsa-mir-133b	HMDD, dbDEMC
22	hsa-mir-15a	HMDD, dbDEMC	47	hsa-mir-142	HMDD, dbDEMC
23	hsa-mir-143	HMDD, dbDEMC	48	hsa-mir-195	HMDD, dbDEMC
24	hsa-mir-27a	HMDD, dbDEMC	49	hsa-mir-15b	dbDEMC
25	hsa-mir-31	HMDD, dbDEMC	50	hsa-let-7d	HMDD, dbDEMC

**Table 4:** The top 50 esophageal neoplasms-related miRNAs candidates predicted by KATZNCP with removed all known esophageal neoplasms-miRNAs associations and the confirmation of these associations

Rank	miRNA name	Evidences	Rank	miRNA name	Evidences
1	hsa-mir-21	HMDD, dbDEMC	26	hsa-mir-27a	HMDD, dbDEMC
2	hsa-mir-146a	HMDD, dbDEMC	27	hsa-mir-146b	dbDEMC
3	hsa-mir-155	HMDD, dbDEMC	28	hsa-mir-133b	dbDEMC
4	hsa-mir-125b	HMDD, dbDEMC	29	hsa-mir-10b	HMDD, dbDEMC
5	hsa-mir-126	HMDD, dbDEMC	30	hsa-mir-142	dbDEMC
6	hsa-mir-145	HMDD, dbDEMC	31	hsa-mir-34c	HMDD, dbDEMC
7	hsa-mir-221	dbDEMC	32	hsa-mir-18a	dbDEMC
8	hsa-mir-16	dbDEMC	33	hsa-mir-101	HMDD, dbDEMC
9	hsa-mir-200c	HMDD, dbDEMC	34	hsa-mir-375	HMDD, dbDEMC
10	hsa-mir-34a	HMDD, dbDEMC	35	hsa-let-7b	HMDD, dbDEMC
11	hsa-mir-31	HMDD, dbDEMC	36	hsa-mir-107	HMDD, dbDEMC
12	hsa-mir-200b	Unconfirmed	37	hsa-mir-9	dbDEMC
13	hsa-let-7a	HMDD, dbDEMC	38	hsa-mir-182	HMDD, dbDEMC
14	hsa-mir-20a	HMDD, dbDEMC	39	hsa-mir-223	HMDD,dbDEMC
15	hsa-mir-196a	HMDD, dbDEMC	40	hsa-mir-210	HMDD,dbDEMC
16	hsa-mir-218	dbDEMC	41	hsa-mir-34b	HMDD, dbDEMC
17	hsa-mir-1	dbDEMC	42	hsa-mir-181a	dbDEMC
18	hsa-mir-17	dbDEMC	43	hsa-mir-24	dbDEMC
19	hsa-mir-222	dbDEMC	44	hsa-let-7d	dbDEMC
20	hsa-mir-200a	HMDD, dbDEMC	45	hsa-mir-92a	HMDD, dbDEMC
21	hsa-mir-29a	dbDEMC	46	hsa-mir-133a	HMDD,dbDEMC
22	hsa-mir-143	HMDD, dbDEMC	47	hsa-mir-205	HMDD, dbDEMC
23	hsa-mir-148a	HMDD, dbDEMC	48	hsa-mir-183	HMDD, dbDEMC
24	hsa-mir-124	HMDD, dbDEMC	49	hsa-let-7i	dbDEMC
25	hsa-mir-199a	HMDD, dbDEMC	50	hsa-mir-125a	dbDEMC

## Discussion and conclusion

Considerable studies have shown that miRNAs play an important role in a wide range of biological processes. miRNAs are associated with the occurrence and development of many complex diseases. Many miRNAs are considered as the ideal biomarkers for disease prevention, diagnosis, and treatment. Given the time consumption and intensive labor to verify the association between miRNA and disease through traditional biological experiments, the prediction of the potential association between miRNA and disease through computational methods as an effective supplement to biological experiments has become a hot topic in bioinformatics.

In this paper, a new prediction model KATZNCP was proposed, which consisted of three stages: constructing accurate similarity network, obtaining miRNA–disease prediction score by KATZ algorithm, and obtaining two miRNA–disease refinement score by network consistency projection. Reasonable construction of the similarity relationship between disease and miRNA can improve the prediction accuracy of the computational method. In constructing a reasonable similarity relationship, Gaussian kernel function was applied to the topological association relationship network among biological information nodes. The similarity of Gaussian kernel spectrum between diseases and miRNAs was calculated by experimentally verifying disease–miRNA association information. Then, an accurate disease similarity network was constructed by integrating the experimentally verified disease-miRNA association information, semantic similarity network among diseases, and Gaussian interaction profifile kernel similarity information among diseases. An accurate miRNA similarity network was constructed by integrating the experimentally verified disease–miRNA association information, the functional similarity network among miRNAs, and the Gauss kernel similarity among miRNAs. Afterward, the integrated disease similarity network, the integrated miRNA similarity network, and the known miRNA–disease association were used to construct a heterogeneous network. The KATZ algorithm was applied on the heterogeneous network to obtain the initial association score between miRNA and diseases. The calculated association scoring network of the initial score was projected into the integrated disease similarity network and integrated miRNA similarity network to obtain the consistency information among vectors. Then, the consistency projection scoring matrix based on the disease space and miRNA space was obtained. Finally, the two consensus prediction scores were weighted as the final miRNA–disease association prediction score. The prediction model algorithm was simple in design and low in time complexity, and it can be applied to the prediction of isolated diseases and new miRNAs. Given the local information obtained in heterogeneous networks through KATZ and the global information among the experimentally verified disease–miRNA association network, the integrated miRNA similarity network, and the integrated disease similarity network obtained through the consistency projection, the prediction results were ensured to be unbiased to the miRNA with more known associations (Additional file 1, Additional file 2, Additional file 3).

In the case study, lung neoplasms and esophageal neoplasms were selected for experimental study. Among the top 50 miRNA prediction related to corresponding diseases, the validation accuracy in HDMM3.2 and dbDEMC databases was 100% and 94%, respectively. For the prediction of isolated disease cases, 100% and 98% of the top 50 miRNAs were confirmed by the two above mentioned databases. For some miRNAs without experimental verification, relevant correlation evidence was found in recent literature. The reliable prediction of KATZNCP provided insight into the identification of potential miRNA biomarkers and contributed to the future work on the involvement of miRNA in human disease mechanisms.

## Supplementary Information

**Additional file 1**. Known miRNA-disease associations.

**Additional file 2**. diseases_list.

**Additional file 3**. miRNAs_list.

## Data Availability

All datasets generated for this study are included in the article/Supplementary Material.
